# Quantum interference measurement of spin interactions in a bio-organic/semiconductor device structure

**DOI:** 10.1038/srep09487

**Published:** 2015-03-30

**Authors:** Vincent Deo, Yao Zhang, Victoria Soghomonian, Jean J. Heremans

**Affiliations:** 1 Physics Department, Ecole Polytechnique, 91128 Palaiseau, France; 2 Department of Physics, Virginia Tech, Blacksburg VA 24061, USA

## Abstract

Quantum interference is used to measure the spin interactions between an InAs surface electron system and the iron center in the biomolecule hemin in nanometer proximity in a bio-organic/semiconductor device structure. The interference quantifies the influence of hemin on the spin decoherence properties of the surface electrons. The decoherence times of the electrons serve to characterize the biomolecule, in an electronic complement to the use of spin decoherence times in magnetic resonance. Hemin, prototypical for the heme group in hemoglobin, is used to demonstrate the method, as a representative biomolecule where the spin state of a metal ion affects biological functions. The electronic determination of spin decoherence properties relies on the quantum correction of antilocalization, a result of quantum interference in the electron system. Spin-flip scattering is found to increase with temperature due to hemin, signifying a spin exchange between the iron center and the electrons, thus implying interactions between a biomolecule and a solid-state system in the hemin/InAs hybrid structure. The results also indicate the feasibility of artificial bioinspired materials using tunable carrier systems to mediate interactions between biological entities.

In a hybrid bio-organic/semiconductor lithographic structure, quantum interference experiments are used to study spin interactions between the iron center in hemin and a proximate two-dimensional electron system (2DES) at the surface of InAs. Hemin (Fig. 1a) is an iron porphyrin similar to the prosthetic heme group in hemoglobin, where the iron center impacts biological functions. In the hemin/semiconductor structure a magnetic characterization method is employed deriving its sensitivity from electrically-measured quantum interference, evidenced as electron antilocalization^1^, and from the engineered nanoscale proximity between the 2DES to the local spin moments in hemin. Hemin influences the spin environment of the electrons, resulting in a temperature-dependence of the electron spin-flip scattering. Here we demonstrate that the influence of the spin environment on the decoherence times of low-dimensional electrons can be electronically measured in a bio-organic/semiconductor device to characterize a biomolecule. The approach is in principle similar to magnetic resonance techniques in its use of decoherence times, but is implemented electronically.

The 2DES in this study is the electron accumulation layer at the surface of (001) InAs, schematically represented in Fig. 2a^2^,^3^. The Rashba spin-orbit interaction (SOI)^4^,^5^ in this 2DES enables our method. Metalloporphyrins, cyclic π-conjugated molecules with a centrally hosted metal ion, are actively studied in fields from optoelectronics to spin electronics and sensing, due to the richness of phenomena arising from the interaction of the metal ion and the π-system. Of special biological interest are metalloporphyrins containing iron, such as hemoglobin, myoglobin, and cytochromes, all containing a heme moiety. The spin and local magnetic moment of the iron centers in heme imbue the functional group with many of its properties^6^, and can be used to determine the state of the ion^7^,^8^,^9^,^10^. The biophysics interest in the iron centers' spin states and their impact on biological functions is substantial, long-standing^11^,^12^ and ongoing. Hemin-functionalized InAs and InP surfaces *e.g.* act as sensitive sensors for NO^13^. The electronic structure of iron in hemoglobin and hemin in solution indicate a high-spin ferric state^14^. First-principles atomistic calculations^15^ of myoglobin bound with different ligands suggests fluctuating magnetic moments in the heme portion, correlated with high- and low-spin states of the iron center. In this work, the labile state of the biologically relevant spin in hemin opens avenues for measuring the spin exchange with nearby electrons in a nanoscale solid-state hybrid device, thereby quantifying the spin state. Like studies may moreover provide the insight to construct bioinspired nanostructured materials using tunable electron systems as intermediary to mediate an exchange between biological entities.

The spin interactions measurably modify the quantum corrections to the 2DES electrical conductivity at low temperatures (< 10 K). The corrections in electronic transport stem from quantum interference of electron partial waves on time-reversed pairs of backscattered trajectories. Constructive interference results in increased backscattering and hence increased resistance (weak localization, WL). Under SOI a closed diffusive path is accompanied by a spin rotation of 2π, resulting in a change of sign of the wavefunction, destructive interference, reduced backscattering and hence decreased resistance (antilocalization, AL)^16^. AL thus originates in spin-dependent interference of electrons and is sensitive to spin decoherence^1^,^16^,^17^,^18^,^19^. The spin decoherence of the surface electrons in turn is a sensitive gauge of their spin interactions with magnetic impurities, exceeding direct magnetic measurements in sensitivity^20^ and capable of quantifying spin interactions in our low-dimensional spin system. Parallels with magnetic resonance methods (EPR and NMR) can be found in the method of characterizing the local spin environment by measuring the spin decoherence time (a T_2_ time). In AL, a magnetic field *B* applied normally to the 2DES breaks the time reversal symmetry and reduces the interference effect, leading to a characteristic magnetoresistance (MR)^1^,^17^,^18^,^19^ determined by four characteristic decoherence or scattering rates^1^,^19^,^21^. The scattering rates (inverse scattering times) are the elastic scattering rate τ_0_^−1^ as independently deduced from the areal electron density n_s_ and from the electron mobility, the inelastic scattering rate τ_i_^−1^, the SOI scattering rate τ_SO_^−1^, and the magnetic spin-flip scattering rate τ_s_^−1^. The total electron decoherence rate, τ_φ_^−1^, is obtained as τ_φ_^−1^ = τ_i_^−1^ + 2 τ_s_^−1^. The spin-flip rate τ_s_^−1^ is here of particular value because it conveys information about the interactions between the surface hemin and the 2DES^1^,^19^. The experiments measure the longitudinal resistance *R*(*B*), with data presented as MR, Δ*R*(*B*) = *R*(*B*) − *R*(*B* = 0) normalized to *R*_0_ = *R*(*B* = 0). Since the quantum corrections to the two-dimensional conductivity *σ*_2_(*B*) are small, we have Δ*R*(*B*)/*R*_0_ ≈ − Δ*σ*_2_(*B*)/*σ*_2_(*B* = 0) where Δ*σ*_2_(*B*) = *σ*_2_(*B*) − *σ*_2_(*B* = 0). This work uses the expression for Δ*σ*_2_(*B*) of Ref. 18 modified for the presence of spin-flip scattering^19^: 
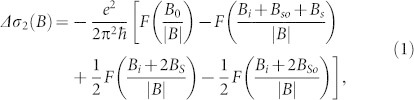
where *F*(*x*) = Ψ(1/2 + *x*) − ln(*x*), Ψ is the digamma function, and each scattering time τ*_α_* (with α = 0, *i, so, s*) enters via a characteristic magnetic field 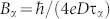, with *D* the two-dimensional diffusion coefficient. A numerical fit of Δ*R*(*B*)/*R*_0_ to equation (1) allows for the extraction of the unknown scattering rates. Expressions derived from Ref. 18 have the advantage of computational simplicity and physical transparency^22^, while assuming a diffusive transport regime, where also the Elliott-Yafet (EY) spin-orbit scattering mechanism^23^,^24^ dominates over the D'yakonov-Perel (DP) mechanism^23^,^24^, and have applicability in the low magnetic field regime with *B* < *B_0_*. As shown below, the data numerically follows Eq. 1 very closely, while yielding a consistent series of scattering times compatible with physical understanding. More important in this method than absolute values of the τ*_α_* is that the relative values of the τ*_α_* do not vary significantly with consistent application of the same model. Then reliable information is obtained from the comparative trend under surface coverage by hemin and under varying temperature *T*. Consistency of the data with the model is further demonstrated by showing, below, that the behavior of SOI scattering rate τ_SO_^−1^ is compatible with the EY mechanism.

## Results

**Figure 1:**
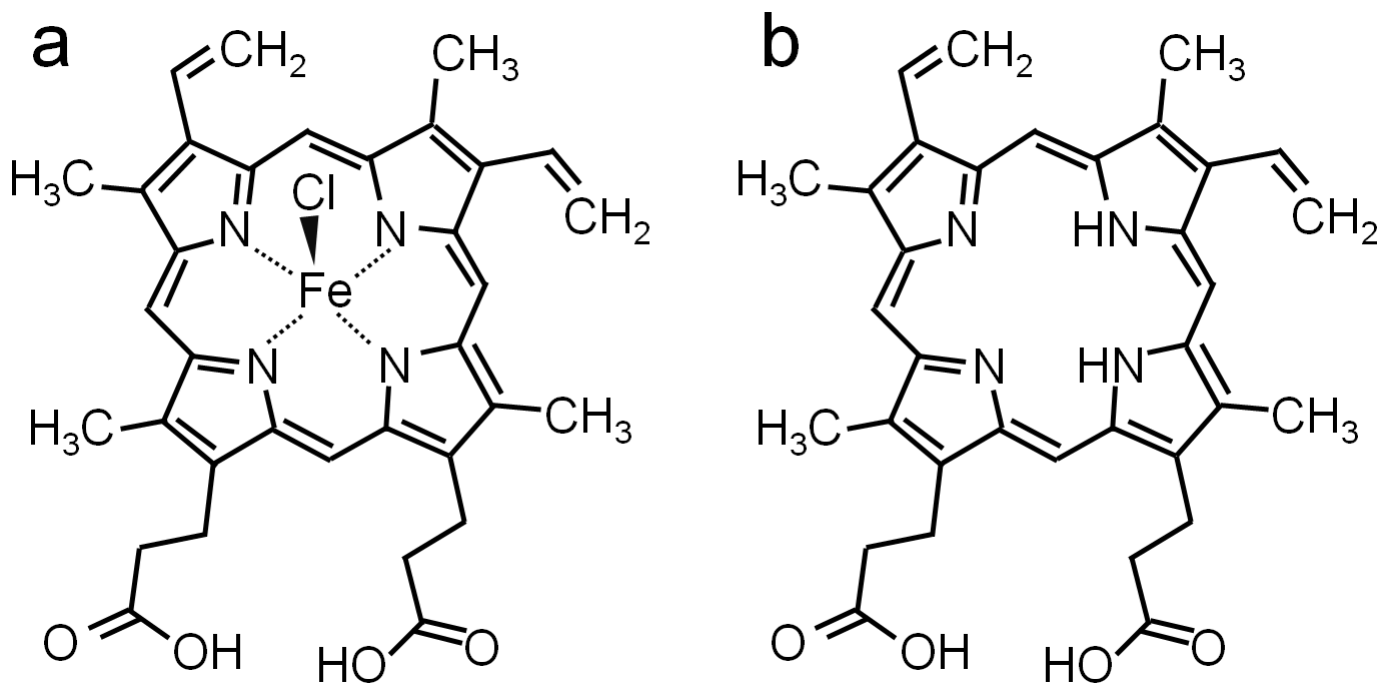
Chemical structures of the surface species used in this work. (a), Structure of hemin chloride (hemin), with the Fe center in the +3 oxidation state. (b), Structure of Protoporphyrin IX (PP-IX).

**Figure 2:**
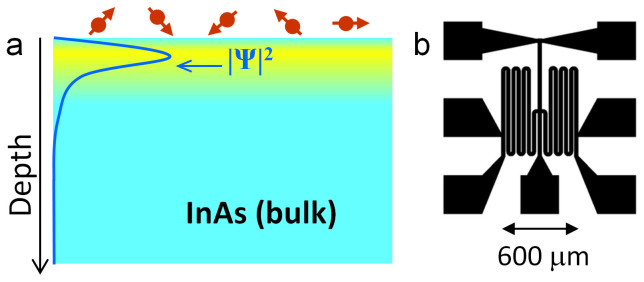
2DES at InAs surface and serpentine pattern. (a), Schematic cross-section of the 2DES electron accumulation layer (~20 nm in depth) at the surface of InAs. |Ψ|^2^ represents the electron wavefunction probability density (arbitrary units), maximal ~ 8 nm from the surface, and tailing off into the InAs bulk. The hemin molecules on the surface are represented by red circles, and the spin associated with them as red arrows. (b), Optical photograph of a serpentine pattern, with electrical contact areas. Serpentine mesas are fabricated by photolithography, and are employed to increase the MR signal by increasing the InAs channel length to width ratio.

Hemin is used as a model for hemoglobin, with protoporphyrin IX (PP-IX, Fig. 1b), as the iron-free species. Comparative quantum interference experiments^19^ involving PP-IX, hemin, and solvent only, are performed to assess the influence of the iron center. The InAs surface is patterned with serpentine mesas (Fig. 2b) (Supplementary Section 1). Starting with three serpentines, we deposit 0.1 μL of 6 μM solution of PP-IX on one, and of hemin on the second, both in the same ethanol : dichloromethane solvent. The third serpentine is covered by 0.1 μL solvent only, as reference for the comparative study. As the PP-IX and hemin solutions air-dry, they leave behind multilayers of PP-IX or hemin on their respective serpentine. After drying, the three serpentines are cooled-down to measurement temperatures *T* (0.4 K ≤ *T* ≤ 5 K) at the same time, minimizing cool-down variations. The AL signals are comparatively measured on the three serpentines, and it is from the comparative (rather than absolute) data that conclusions are drawn about the interactions between hemin spins and electron spins. Comparative measurements between serpentines fabricated in parallel and in identical conditions, and with the same processing and cool-down, eliminate variations due to the sensitivity of the InAs 2DES to external factors.

Surface characterization of hemin-functionalized InAs for NO sensing^13^ and surface studies of PP-IX on Pt *vs* ZnPP-IX on Pt^25^ suggest that PP-IX and hemin anchor to a metallic surface through the carboxylic groups, predominantly in an upright orientation for PP-IX, although for ZnPP-IX, the molecules were at an angle from the normal to the surface. Several interactions are at play, including chemisorption via the carboxylic groups, π-interaction between protoporphyrins, and electrostatic interaction between the metal ion of the hemin and the 2DES. Atomic force micrographs (Supplementary Section 2) of multilayers of PP-IX and hemin on unpatterned InAs surfaces show differences in aggregation patterns, presumably due to various interactions of the molecules and the surface. The quantum interference measurement is an averaging technique on an ensemble of surface magnetic moments and hence minimizes effects of local differences in n_s_, surface attachment and orientation.

**Figure 3:**
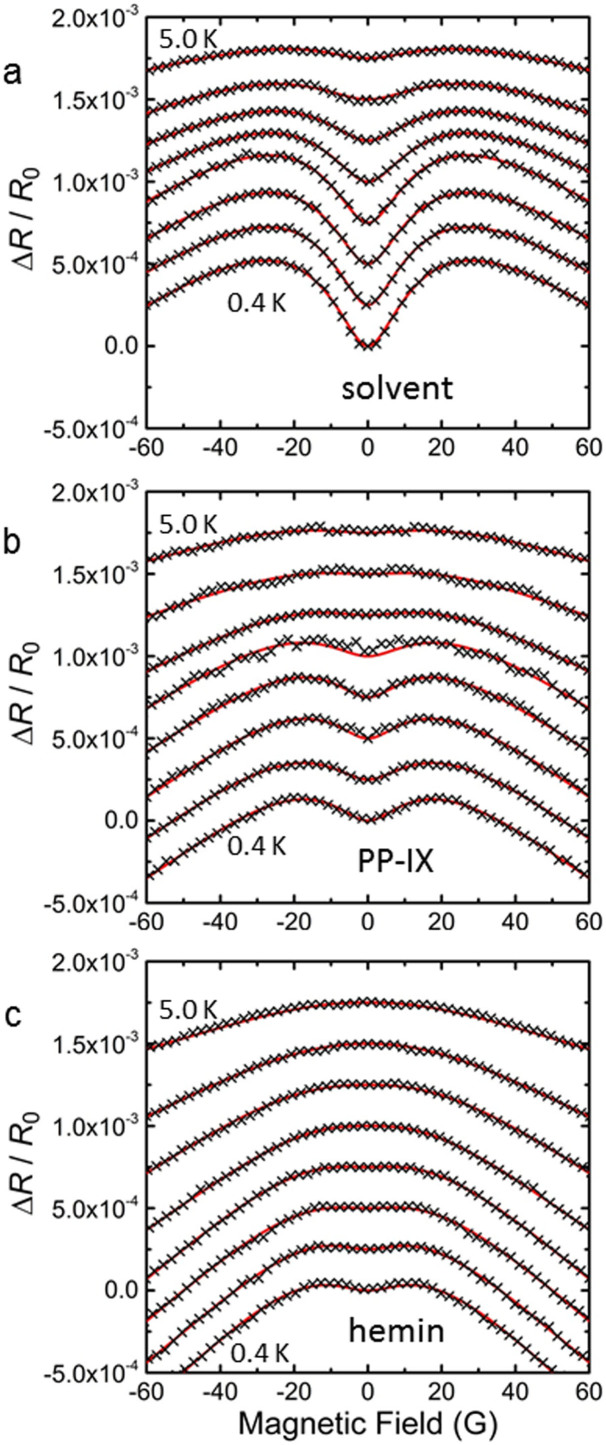
Magnetoresistance due to AL for the three serpentines as Δ*R*(*B*)/*R*_0_, parametrized in *T*. (a), Solvent-covered serpentine. (b), PP-IX-covered serpentine. (c), Hemin-covered serpentine. In each panel the 8 curves are offset by 2.5 × 10^−4^. Each curve represents data at different *T*; from low to high, *T* = 0.4, 0.55, 0.70, 0.85, 1.2, 2.0, 3.0, 5.0 K. Black crosses represent experimental data, out of which 1 in 10 is plotted for clarity. Solid red lines are best numerical fits to equation (1).

**Figure 4:**
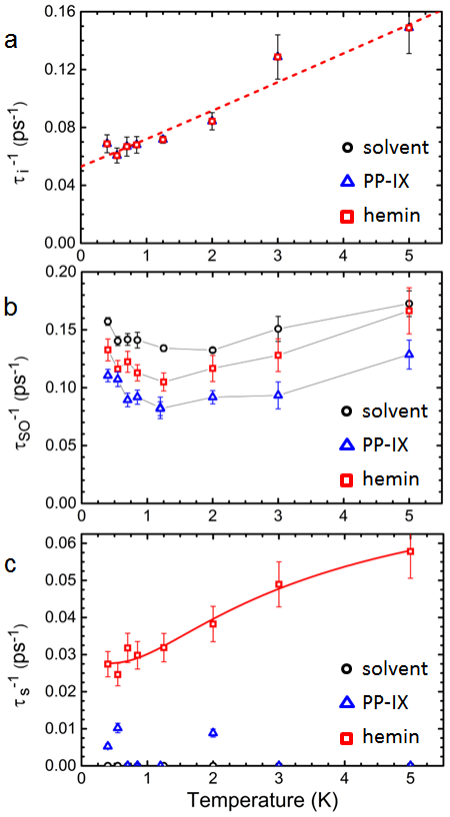
Scattering rates as function of *T*. (a), The inelastic scattering rates τ_i_^−1^. (b), The SOI scattering rates τ_SO_^−1^. (c), The magnetic spin-flip scattering rates τ_s_^−1^. In each panel black circles represent scattering rates for the solvent-covered serpentine, blue triangles for the PP-IX-covered serpentine, and red squares for the hemin-covered serpentine. In (a) the dashed red line represents a best fit to τ_i_^−1^ ~ *T*^1.02^, and in (c) the solid red line represents a best fit to τ_s_^−1^ = τ_Δs_^−1^ + (τ_tf_^−1^) exp(-*E_a_*/*k_B_T*), both as discussed in the text. In (b) the lines connecting data for τ_SO_^−1^ form guides to the eye only. Error bars are indicated by vertical lines.

Figures 3a-c contain MR data due to AL for the three serpentines, presented as Δ*R*(*B*)/*R*_0_. Data are parametrized in *T*, with 0.4 K ≤ *T* ≤ 5.0 K. At lower *T* the positive MR for *B* ≈ 0 crosses over to negative MR at higher *B*, a characteristic of AL due to SOI beneficial to obtain values for τ_s_^−1^ since the non-monotonous MR permits unambiguous numerical fits between data and equation (1). The AL correction fades at higher *T* due to an increased phase decoherence (quantified below). The data in Figs. 3a–c differ markedly, demonstrating the dependence of AL on specific surface species. The numerical fits of the data in Fig. 3 to equation (1) were realized using the Monte-Carlo based cross-entropy method^26^,^27^ (Supplementary Section 4), which is well-adapted to non-linear multi-extremal optimization problems in a multi-parameter space. Figure 3 shows an excellent correspondence between the data and Eq. 1. The fitting parameters are *B_i_*, *B_so_* and *B_s_* (proportional to the corresponding scattering rates), while the elastic scattering field *B*_0_ is determined from the measured n_s_ and mobility in the 2DES (Methods, and Supplementary Section 3). Uniqueness of the numerical fits is aided by the characteristic crossover from positive MR to negative MR, here occurring for *B* ≈ 20 G. Equation 1 assumes a range of *B* not exceeding *B*_0_. From the transport parameters (Supplementary Section 3), the average of *B*_0_ over serpentines and over *T* can be estimated as *B*_0_ ≈ 32 G. Thus the range of *B* most determining for the fits is included in theoretical region of validity of Eq. 1, supporting the consistency between model and data. In Eq. 1 *B_i_*, *B_so_* and *B_s_* appear as combinations *B_so_* − *B_s_* (corresponding to an effective spin scattering rate) and *B_i_* +2 *B_s_* (corresponding to τ_φ_^−1^) such that only two of the unknown fields can be determined from one data set and an added constraint is necessary^19^,^28^. We consider that *B_s_* = 0 for the reference solvent-covered serpentine, where no magnetic scattering centers are introduced on the 2DES, and therefore values for *B_i_* and *B_so_* can be determined. For PP-IX- and hemin-covered serpentines, we assume that τ_i_^−1^ (from which *B_i_* is determined) is unchanged from the solvent-covered serpentine. These assumptions have precedents in the literature^1^,^19^,^28^ and are justified from the knowledge that in metal systems magnetic impurities lead to an increase in magnetic spin-flip and SOI scattering rates but leave inelastic scattering unaffected. The identical processing and cool-down history of the serpentines helps validate this assumption, as does the resulting excellence of the fits. With *B_i_* provided by the reference measurement, *B_so_* and *B_s_* can be determined for the PP-IX- and hemin-covered serpentines to determine the change induced in spin-orbit and spin-flip scattering by the addition of the surface species. Below we discuss the various extracted scattering rates *vs* *T*, all presented in Fig. 4. Error bars in Fig. 4 take into account the sensitivity of the fitting residue to a variation in the *B_α_*.

## Discussion

We start with the inelastic scattering rates τ_i_^−1^ (Fig. 4a) which show a dependence on *T* close to linear. By the procedure above, the values of τ_i_^−1^ are unchanged between the PP-IX-, hemin-, and solvent-covered serpentines. The data can be fitted to τ_i_^−1^ = A*T*^1.02^ + τ_sat_^−1^ over the measurement range (A and τ_sat_^−1^ are fitting parameters). For low-dimensional systems at low *T*, Nyquist decoherence^21^,^29^ originating in fluctuations in the electromagnetic background often determines τ_i_^−1^, and in two dimensions results in τ_i_^−1^ ~ *T*, consistent with the present measurements. The term τ_sat_^−1^ denotes a low-*T* saturation of phase coherence, frequently observed while not fully understood^21^. In this measurement τ_sat_ ≈ 19 ps, constituting an upper bound for τ_i_.

The spin-orbit scattering rates τ_SO_^−1^ contained in Fig. 4b convey the relative strength of SOI among the differently-covered serpentines. Within the error bars, the non-monotonic dependence on *T* tracks the dependence of n_s_ and mobility in the 2DES (Supplementary Section 3) and can be used to qualitatively identify the dominant spin-orbit scattering mechanism. In the EY mechanism a momentum scattering event leads to a spin-orbit scattering event due to spin-momentum locking, and a dependence τ_SO_^−1^ ~ *E_F_*^2^/τ_0_ is expected^23^,^24^, where *E_F_* denotes the Fermi level. Hence in a 2DES with constant density-of-states, the EY mechanism leads to τ_SO_^−1^ ~ n_s_^2^/τ_0_. In the DP mechanism spin-orbit scattering is mitigated by motional narrowing and τ_SO_^−1^ ~ n_s_^3^ τ_0_ is expected^23^,^24^. Experimentally, minima are observed in τ_SO_^−1^, n_s_ and τ_0_, at *T* ≈ 1.2...2 K, and the highest measured values occur at *T* ≈ 5 K. The ratio between low and high values for n_s_^2^/τ_0_ is approximately 0.60, for n_s_^3^ τ_0_ approximately 0.35, and for τ_SO_^−1^ approximately 0.69. From the ratios it is apparent that the EY mechanism provides a better description for spin-orbit scattering in this system than does the DP mechanism, as expected for a system where mobility is adversely impacted by its proximity to an exposed surface. The EY mechanism limiting τ_SO_^−1^ is consistent with the use of Eq. 1. With the EY mechanism limiting τ_SO_^−1^, it is further expected that *B_so_* will be less sensitive to the surface electric field^24^, an advantage for the present method. Indeed in the InAs surface system the electric field is not well controlled and a large randomness in the combination *B_so_* − *B_s_*, from which the magnetic spin-flip rate τ_s_^−1^ is determined, would also have induced additional uncertainty in τ_s_^−1^.

Compared to the solvent-covered serpentine, PP-IX and hemin reduce τ_SO_^−1^ and concomitantly reduce the SOI experienced in the 2DES, by ~ 35% for PP-IX and ~ 15% for hemin. The relative reduction in τ_SO_^−1^ for PP-IX and hemin is maintained over the range of *T* and hence consistently points to an influence of surface coverage. The origin of the decrease must be searched for in the electrostatic interactions between the surface entities and the metallic 2DES. The delocalized electrons associated with the cyclic conjugated groups in the PP-IX molecules are expected to induce a shielding of the surface electric field, diminishing inversion asymmetry and SOI. The same effect will occur under hemin coverage, with same overall organic structure as PP-IX. However, the presence of the Fe center of higher atomic weight will increase the average SOI experienced by electrons in its proximity, an effect also observed in metals systems with adatoms^19^,^30^. The effect will diminish the reduction in SOI relative to PP-IX, as indeed observed.

Figure 4c contains the magnetic spin-flip rates τ_s_^−1^. Within the error bars, the data show that spin-flip scattering is low in the PP-IX-covered serpentine and substantial in the hemin-covered serpentine where furthermore a distinct *T*-dependence is observed. The low values of τ_s_^−1^ for PP-IX coverage can be understood from the lack of magnetic moment in the molecule. The origin of magnetic spin-flip scattering indeed lies in spin-spin interaction between magnetic moments and the electron spin. An important difference derives from the Fe center in hemin, where a spin exchange mechanism inducies substantial spin-flip scattering in the 2DES. The magnitude and *T*-dependence of τ_s_^−1^ implies an interaction **σ.S** between the hemin local moments **S** and electrons spin **σ**, with **S** denoting the total spin of the hemin's Fe^+3^ ion 3d electrons. The existence of spin-flips in the 2DES moreover implies a change in **S**. In hemin's Fe^+3^ ion, the spin state of the 3d electrons is affected by the surrounding ligands. A strong-field ligand favors pairing of the electrons to form a low-spin S = 1/2 state (^2^T_2g_) while a weak-field ligand favors unpaired electrons forming a high-spin S = 5/2 state (^6^A_1g_)^7^,^9^,^31^. For a given Fe^+3^ in hemin, different near-degenerate spin states can occur, resulting in thermal mixtures^8^,^10^,^15^,^31^,^32^. Moreover, quantum-mechanical mixed-spin ground states are also possible^9^. Further, the magnetic susceptibility of polycrystalline hemin has been observed to follow ferromagnetic behavior, with a Curie-Weiss temperature ~ 32 K, although fully developed magnetization and long-range order were not observed^31^. Since the Curie-Weiss temperature is well above the present measurement range, paramagnetic behavior may be excluded as the origin for the *T*-dependence of τ_s_^−1^. A more likely origin lies in the occurrence of two labile spin states, S = 1/2 and 5/2, observed previously^32^, due to ligand effects in the solid-state environment possibly fluctuating and leading to transiently quasi-degenerate spin states. Spin exchange interactions with the InAs surface electrons then mediate transitions between finely split energy states of the Fe^+3^ ion, leading to the observed increase in spin-flip scattering over that observed for PP-IX coverage, and to the *T*-dependence of τ_s_^−1^. Although the detailed physics differs, the process can be mapped on a model^33^ for the rate of magnetization reversal of 2-state superparamagnetic particles with an anisotropy energy barrier (Néel-Brown expression^34^), leading to an expression τ_s_^−1^ = τ_Δs_^−1^ + (τ_tf_^−1^) exp(-*E_a_*/*k_B_T*), where in this case *E_a_* denotes the average energy difference between the system with Fe^+3^ in the low-spin *vs* high-spin state, τ_tf_^−1^ is the transition frequency, and τ_Δs_^−1^ is included to take into account any *T*-independent spin-flip rate of processes unrelated to the Fe^+3^ mixed spin state. The hemin data can be fitted to the above expression over the measurement range, as depicted in Fig. 4c, and the resulting parameters acquire reasonable values: *E_a_* ≈ 0.27 meV, τ_tf_^−1^ ≈ 0.057 ps^−1^, and τ_Δs_^−1^ ≈ 0.028 ps^−1^. The low *E_a_* indeed indicates near-degenerate levels, while τ_tf_^−1^ of the same approximate magnitude as the actually measured τ_s_^−1^ validates the physical picture, and the low τ_Δs_^−1^ implies that the labile spin state of Fe^+3^ plays an important role in the 2DES spin flip scattering. The increase in τ_s_^−1^ in the presence of hemin compared to solvent or PP-IX indicates the existence of spin interaction between the Fe^+3^ and the 2DES, serving as example of an artificially constructed structure where a nanoscale solid-state system and a biologically relevant moiety interact.

To conclude, in a bio-organic/semiconductor device a quantum interference measurement was used to quantify the spin interactions between a low-dimensional solid-state electron system and hemin. Antilocalization as a probe of spin decoherence allowed measurements akin to magnetic resonance techniques. The study points towards more active hybrid systems, where bioinspired functional hybrid materials use quantum-mechanical paths available in solid-state systems to achieve interactions between proximate biological entities.

## Methods

### Sample preparation

All chemicals were used as received. From Sigma-Aldrich, protoporphyrin-IX (PP-IX in text), empirical formula C_34_H_34_N_4_O_4_, product number P8293, and hemin chloride (hemin) from porcine, empirical formula C_34_H_32_ClFeN_4_O_4_, product number 51280, were purchased. Dichloromethane was HPLC grade and obtained from CM Science, while 95% denatured ethanol was purchased from EMD. Serpentine patterns where fabricated by photolithography (Supplementary Section 1). The hybrid bio-organic/semiconductor structures were generated by depositing hemin and PP-IX solution onto serpentine patterns. Equal number of moles of PP-IX and hemin are dissolved in the same solvent which is a 1:1 ethanol : dichloromethane mixture, yielding 6 μM solutions. 0.1 μL each of PP-IX, hemin and solvent only are deposited on 3 serpentines, air-dried, and subsequently cooled down and measured.

### Magnetotransport measurements and analysis

Low temperature magnetotransport measurements were performed in a ^3^He cryogenic system equipped with a superconducting magnet. The system has a base temperature of 0.35 K. Localization measurements were achieved in 4-contact low-frequency ac mode, under low current excitation of 20 nA rms to prevent heating of the electronic system. The InAs samples host two types of carriers, namely surface 2DES electrons and bulk electrons. Surface and bulk electrons' densities and mobilities need to be determined, as the presence of multiple carriers results in a classical background in magnetoresistance that has to be subtracted to yield the localization quantum correction, and as the surface electron density and mobility is required to calculate the elastic scattering field *B*_0_. Measurements of longitudinal and Hall resistance vs *B* allowed this characterization of surface and bulk electrons densities and mobilities over *T* via fitting to a two-carrier model of classical magnetoresistance, as described in Supplementary Section 3.

## Author Contributions

V.D. fabricated the InAs samples, performed the AL measurements, and analyzed the data. Y.Z. aided in the sample fabrication and measurement. V.S. and J.J.H. conceived and designed the experiments, interpreted the results and wrote the manuscript. All authors discussed the results and commented on the manuscript.

## Supplementary Material

Supplementary Information Supplementary info: Quantum interference measurement of spin interactions in a bio-organic/semiconductor device structure
