# Supplementary material for: Quantum interference measurement of spin interactions in a bio-organic/semiconductor device structure
Source: Sci Rep. 2015 Mar 30;5:9487. doi: 10.1038/srep09487 (PMC4377638; doi:10.1038/srep09487)
Supplement: Supplementary Information — Supplementary info: Quantum interference measurement of spin interactions in a bio-organic/semiconductor device structure [file srep09487-s1.doc]

**Supplementary Information**

**Quantum interference measurement of spin interactions in a bio-organic/semiconductor device structure**

Vincent Deo, Yao Zhang, Victoria Soghomonian, Jean J. Heremans*

*Corresponding author: [heremans@vt.edu](mailto:heremans@vt.edu)

**Contents**

S1. Fabrication 2

S2. Atomic force microscopy 3

S3. Magnetotransport properties of the InAs surface and bulk 4

S4. The cross-entropy method 7

**References** 9

**S1: Fabrication**

Serpentine mesas were patterned onto the InAs surface via photolithography. The InAs layer was 3.75 μm thick and was grown on (001) oriented GaAs wafers by metal organic chemical vapor deposition. Small, ~ 2x4 mm^2^, pieces of the wafer are cut and cleaned in trichloroethylene, acetone and isopropyl alcohol for 3 minutes each consecutively, and then blow dried with N_2_. The samples are spin-coated with ma-P 1210 photoresist (micro resist technology GmbH), and prebaked at 378 K for 30 minutes. Fig. 2b (of text) shows a single serpentine, while Fig. S1a shows the photomask pattern used, which has twin serpentines. The twin serpentine pattern is transferred onto the photoresist coated InAs surface using a Karl Suss, model MJB3, mask aligner. After UV exposure, the photoresist is removed by a diluted AZ 351 developer (Clariant Corp.). The InAs layer is etched using H_2_SO_4_:H_2_O_2_:H_2_O (1:8:15), and the chip is thoroughly rinsed in deionized water. The photoresist in the remaining covered areas is removed by three steps of soaking and boiling in acetone. Two sets of twin serpentines were fabricated simultaneously, and wired by InSn solder to a 14-pin Dual-Inline-Package header as pictured in Fig. S1b. Of the 4 serpentines, 3 are used for the simultaneous comparative measurement.

**
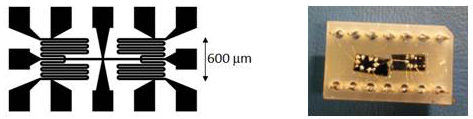
**

**a**

**b**

**Figure S1: Serpentine pair pattern.** **a**, Photomask pattern for a pair of serpentines. **b**, Optical micrograph of two twin serpentines (two chips, left and right) wired-up. Serpentine 1 (left on left chip) is covered with a PP-IX solution, serpentine 2 (right on left chip) with solvent only, and serpentine 3 (right on right chip) with a hemin solution.

**S2: Atomic Force Microscopy (AFM)**


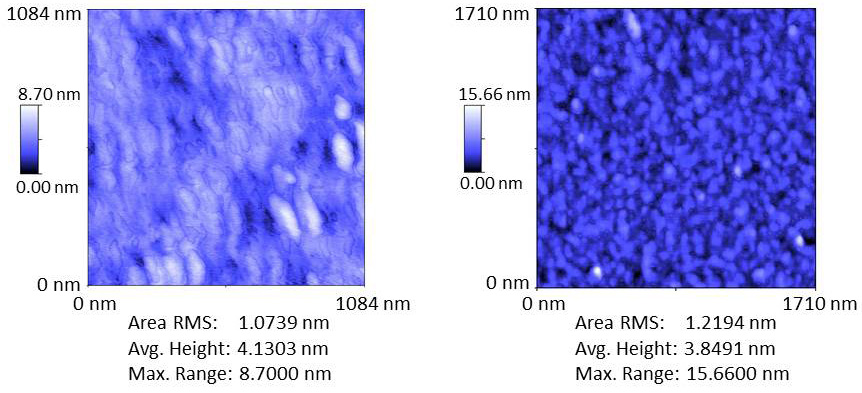
AFM images of hemin- and PP-IX-covered InAs surfaces were obtained by a Veeco Explorer unit, on unpatterned InAs surfaces, from the same wafer and using same concentration

**a**

**b**

**Figure S2: Comparative AFM images on unpatterned InAs surfaces.** **a**, Hemin covered surface. **b**, PP-IX covered surface. In each case, area roughness rms values are indicated, along with the average height and maximum range of heights.

and solvent as used in the localization measurements. We observe qualitative differences between the two samples illustrated in Fig. S2. The PP-IX covered surface appears rougher and with smaller sized aggregates than the surface covered by hemin. Both images in Fig. S2 were obtained under similar conditions and were processed in the same manner.

**S3: Magnetotransport properties of the InAs surface and bulk**

Every serpentine carries contacts for measurement of longitudinal (*R*_xx_) and Hall (*R*_xy_) resistance *vs B*, allowing determination of electron density and mobility values for the InAs surface 2DES over *T*, and allowing subtraction of a classical background contribution to the longitudinal magnetoresistance to highlight the quantum corrections. The presence of 2 types of carriers, namely surface 2DES electrons and bulk electrons, indeed gives rise to a classical background in longitudinal and Hall magnetoresistance, to which, in the case of longitudinal magnetoresistance, the localization quantum correction adds. The determination of surface and bulk electrons densities and mobilities, *n*_s_, *μ*_s_ and *n*_b_, *μ*_b_ respectively, which parametrize the classical background, occurs via fitting of *R*_xx_(*B*) and *R*_xy_(*B*) data to a 2-carrier model of classical magnetoresistance. The determination includes effects of non-parabolicity, yielding for every serpentine an effective mass *m** > 0.024*m*_e_ where *m_e_* is the free electron mass and 0.024*m*_e_ is Γ-point effective mass. Fig. S3 shows, for the solvent covered serpentine at 0.4 K, an example of *R*_xx_ and *R*_xy_ *vs B*, with fitting curves to the 2-carrier model of classical magnetoresistance, and with fitting results.

**
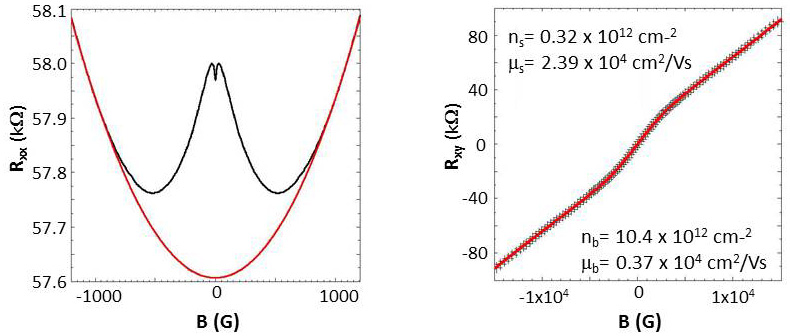
Figure S3: Magnetoresistance measurements for serpentine covered with solvent only at *T* = 0.4 K.** **a**, *R*_xx_ and **b**, *R*_xy_ *vs B* for the solvent-covered InAs serpentine, with the classical part fitted to extract the transport parameters of the 2DES. The black trace in **a** and the black crosses in **b** are experimental points, where in **b** 1 in 30 datapoints is plotted. The red lines in both panels are best fits to the 2-carrier classical magnetoresistance model. Fitting results are indicated in **b**.

**a**

**b**

From the experimental data, the 2DES elastic scattering time τ_0_ = *m***μ*_s_/*e* can be determined, as can the 2DES Fermi wavelength *λ_F_*, Fermi energy *E_F_*, Fermi velocity *v_F_*, and diffusion coefficient, *D* = ½ *v_F_*^2^ τ_0_. From these 2DES parameters the elastic characteristic field *B*_0_ can be calculated. Values for the densities and mobilities of the surface 2DES electrons and bulk electrons, for solvent-, PP-IX-, and hemin-covered serpentines, are obtained for the experiment *T* in the range 0.4 K to 5 K, and the data is presented in Fig. S4. Averages over *T* (0.4 K to 5 K) of the parameters discussed above are presented in Table S1.


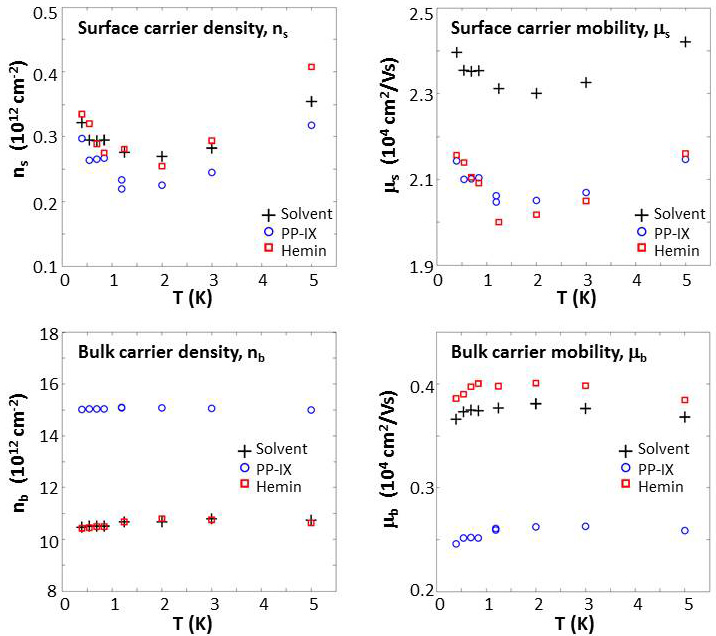


**a**

**b**

**c**

**d**

**Figure S4: Variable temperature areal densities and mobilities of the InAs serpentines.** **a-d,** densities and mobilities of InAs surface 2DES electrons and bulk electrons for all 3 serpentines, covered with solvent (black crosses), or PP-IX (blue circles), or hemin (red squares). **a**, Surface carrier density. **b**, Surface carrier mobility. **c**, Bulk carrier density. **b**, Bulk carrier mobility.

**Table S1: Surface 2DES electron properties**. Areal density *n*_s_, mobility *μ*_s_, diffusion coefficient *D*, ratio of effective mass to free electron mass *m**/*m_e_*, Fermi wavelength *λ_F_*, and Fermi energy *E_F_*, averaged over *T* (0.4 K to 5 K).

| Serpentine | *n_s_* (10^12^ cm^-2^) | *μ_s_* (10^4^ cm^2^/Vs) | *D* (10^2^ cm^2^/s) | 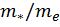 | 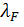(nm) | 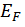(meV) |
| --- | --- | --- | --- | --- | --- | --- |
| Solvent | 0.298 | 2.35 | 9.00 | 0.0512 | 45.9 | 24.1 |
| PP-IX | 0.267 | 2.10 | 7.14 | 0.0499 | 48.5 | 21.7 |
| Hemin | 0.310 | 2.09 | 8.32 | 0.0517 | 45.1 | 25.0 |

**S4: The cross-entropy method**

The cross-entropy (CE) method is a Monte-Carlo based estimation and optimization technique introduced by R. Rubinstein^35,36^, extended to deal with complex multi-extremal optimization by Kroese *et al.*^26^, and applied to AL fitting by Peres *et al.*^27^. The CE method provides a robust and efficient technique to automate the fitting to AL data, using the process of (1) generating starting probabilities, following empirical or theoretical pre-defined criteria, (2) selecting the elite parameters based on a scoring criterion, (3) generating updated samples based on the statistical properties of the latter elite samples, (4) repeating steps 2-3 until a predefined convergence threshold is reached.

In this work multivariate Gaussian probability densities were used to generate parameter sets during the CE processing, for fitting parameters *B_i_*, *B_so_* for the solvent-covered serpentines and *B_so_*, *B_s_* for the hemin- and PP-IX-covered serpentines. The parameter space is probed widely, starting with a logarithmic standard deviation of several decades centered on expected value around 1 G to 10 G. The scoring method uses a least-squares error function over low-field datapoints (*B* < 80 G), with Gaussian ponderation giving more weight to the AL maxima in resistance usually located at *B* < 20 G.

A scale factor in the range 1.2 to 2.8 multiplies the resistance values obtained from equation (1), considerably improving the fit by accounting for geometrical uncertainty. The consistency of the scale factors as a function of temperature *T* is carefully monitored for each serpentine, as is their dynamic behavior during CE iterations. Only slight variations are noted in scattering rates and dependence on *T*, attesting to the self-consistency underlining the validity of the fitting function used in equation 1^19, 33^ and of the approach.

The CE method also allows probing of the fitting error repartition over the parameter space, and allows exploration of the behavior of equation 1 for a particular data set. Fig. S5 shows a contour/density plot of the fitting error depending on characteristic fields *B_i_* and *B_so_* for the solvent-covered serpentine at 0.4 K, and shows the converging behavior of the CE algorithm.


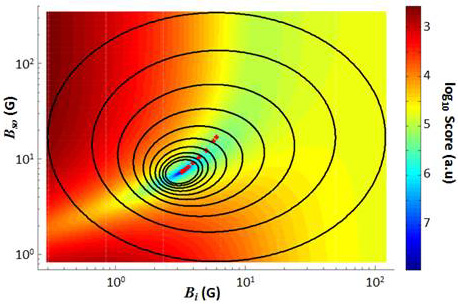


**Figure S5: Density plot of fitting error and convergence of the CE algorithm.** Fit score distribution depending on *B_i_* and *B_so_* values, for AL data of the solvent-covered serpentine at 0.4 K. Colors: density plot of the fitting error depending on characteristic magnetic fields *B_i_* and *B_so_*. Red crosses and black contours: CE algorithm averages and 1-σ domains, from iteration 1 (top right cross, outer ring) to iteration 10 (bottom left cross, inner ring). The best fit occurs with parameters inside the blue spot where the score is maximal and the error is minimal. It is to be noted that final n-σ confidence domains show substantial parameter correlation, with boundaries on the contour lines of the plot.

**References**

35. Rubinstein, R. Y. & Kroese, D. P. *The cross-entropy method: a unified approach to combinatorial optimization, Monte-Carlo simulation and machine learning* (Springer Science +Business Media Inc. USA, 2004).

36. Rubinstein, R. Y. Optimization of computer simulation models with rare events. *Eur. J. Oper. Res.* **99**, 89-112 (1997).

37. Zhang, Yao & Heremans, J. J. Effects of ferromagnetic nanopillars on spin coherence in an InGaAs quantum well. *Solid State Commun.* **177**, 36-41 (2014).
